# The family, school and community linkage of improving adolescent physical fitness and health: based on the ANT

**DOI:** 10.3389/fpsyg.2025.1663693

**Published:** 2025-12-03

**Authors:** Yangyang Liu, Qiuhan Zhu, Shuqing Li, Linhan Yao, Duan Wang

**Affiliations:** 1 School of Kinesiology and Physical Education, Zhengzhou University, Zhengzhou, China; 2 Student Services Department, Hainan Lausanne Tourism University, Sanya, China

**Keywords:** Actor-Network Theory, adolescent physical fitness and health, family school and community, synergetic net, school physical education

## Abstract

**Introduction:**

The issue of insufficient physical activity among adolescents is multifaceted and cannot be effectively addressed by a single organization or department. Therefore, cross-sectoral and inter-organizational collaboration offers a promising approach to meeting the complex needs of adolescents’ early development and physical fitness promotion. Supported by Actor-Network Theory (ANT), this study systematically analyzes how urban China establishes a Family–School–Community (FSC)-centered network for promoting adolescent physical health.

**Methods:**

Through case studies, semi-structured interviews, and participant observations, the study identifies and captures the relationships and categories among various actors (both human and non-human), barriers and interest factors, network entry thresholds and sustainability conditions, as well as the alignment among participants, resources, and institutional support.

**Results:**

Qualitative analysis of the data reveals three core themes: (1) actor identification and determination, (2) network formation through translation, and (3) criteria for actor enrollment and mechanisms for resolving controversies. The findings indicate that cross-sectoral collaboration aimed at promoting adolescents’ physical health is either largely absent or remains fragile, suggesting that local potential has not been fully leveraged. These results highlight the lack of effective actor recruitment within the network-building process.

**Conclusion:**

Consequently, it is essential to mobilize actors, policies, resources, and information exchange platforms to foster adolescents’ participation in physical activities and to strengthen cross-sectoral cooperation policies and practices that support their healthy development.

## 1 Introduction

The widespread prevalence of unhealthy lifestyles characterized by physical inactivity and sedentary behavior has become a pressing public health concern (Althoff et al., 2017). These behaviors are closely associated with a wide range of adverse health outcomes. Notably, insufficient physical activity has been identified as the fourth leading risk factor for global mortality. In 2016, the prevalence of insufficient physical activity among adolescents worldwide reached 81.0% (Guthold et al., 2020). Although the role of school-based physical education in promoting children’s and adolescents’ physical health has been increasingly recognized in recent years, it has not effectively curbed the persistent problems of physical inactivity and sedentary lifestyles within this age group. In China, only a small proportion of adolescents meet the recommended standards for physical activity; fewer than 30% of those aged 9 to 18 achieve the national health benchmarks (Zhu et al., 2017). Over the past decade, key indicators of physical activity among Chinese adolescents have shown a significant decline (Huang et al., 2023; Zhang et al., 2021), remaining well below the levels observed in developed countries (Liu et al., 2019). The COVID-19 pandemic has further exacerbated this situation, leading to a global decrease in physical activity and an increase in sedentary behaviors (Hall et al., 2020).

Addressing these challenges requires coordinated efforts across multiple sectors of society. A government-led collaborative mechanism involving families, schools, and communities (FSC) is essential for promoting adolescents’ regular participation in physical activity and improving their overall health (Chen et al., 2019). Establishing such a framework represents a critical strategy for addressing the societal issues associated with insufficient physical activity and sedentary lifestyles among adolescents.

Adolescents’ physical health serves as a vital indicator of social progress and cultural development (Viner et al., 2012). The promotion of adolescent physical health is characterized by multi-actor participation, multidimensional governance, and comprehensive social benefits (Hunt et al., 2015). Although policies have repeatedly advocated for multi-stakeholder collaboration, the lack of concrete implementation strategies—combined with challenges such as fragmented cooperation, unclear boundaries of responsibility, and unequal distribution of power and interests—has hindered the effective participation of community actors such as families and local organizations. Within this context, schools have assumed a leading role in mobilizing external forces to participate in adolescent education and health promotion. However, the top-down approach, in which schools attempt to extend participation initiatives to other social entities, has proven to be largely ineffective (Weiss et al., 2010). While this asymmetric model of collaboration may stimulate a sense of responsibility among core actors, it also constrains the depth and breadth of external engagement in school-based physical education development.

Moreover, the disparities in power, status, and resource allocation between schools and social organizations often lead to conflicts of interest—particularly between families and schools—manifested in weak willingness to cooperate, frequent power disputes, and imbalances between rights and responsibilities (Lam et al., 2016). Consequently, the collaborative momentum behind adolescent physical health and sports initiatives continues to weaken (Davison and Lawson, 2006). It is therefore imperative to construct a practical and integrated coordination model (Li and Moosbrugger, 2021) that effectively guides FSC collaboration and mitigates the ongoing decline in adolescents’ physical health.

Against this background, the present study examines FSC collaboration within social protection networks involved in promoting adolescents’ physical health and development in selected regions of China. The aim is to capture the relational structure, the complexity of interaction patterns, and the quality of support embedded in these existing networks. Guided by the principles of Actor-Network Theory (ANT), this study posits that a deeper understanding of network interactions can inform interventions to strengthen family and school support, thereby reducing social inequalities and improving resource utilization. Furthermore, the findings are expected to reveal both the strengths and weaknesses of relational dynamics within FSC networks, identifying opportunities to enhance coordinated actions for adolescent physical development.

Specifically, this study addresses the following three research questions:

- Q1: Who are the key human and non-human actors in FSC collaborations aimed at promoting adolescents’ physical health in China, and what unique characteristics do these actors exhibit within the Chinese sociocultural context?
- Q2: Under China’s institutional constraints and cultural norms, through what mechanisms do these heterogeneous actors achieve effective collaboration and linkage to jointly enhance adolescents’ physical health?
- Q3: Which actors play central roles in the translation process of the collaborative network, and how can the translation mechanisms, aligned with China’s policies and social realities, ensure the stable operation of the network?

## 2 Research background

### 2.1 Theoretical foundation

**Figure 1:**
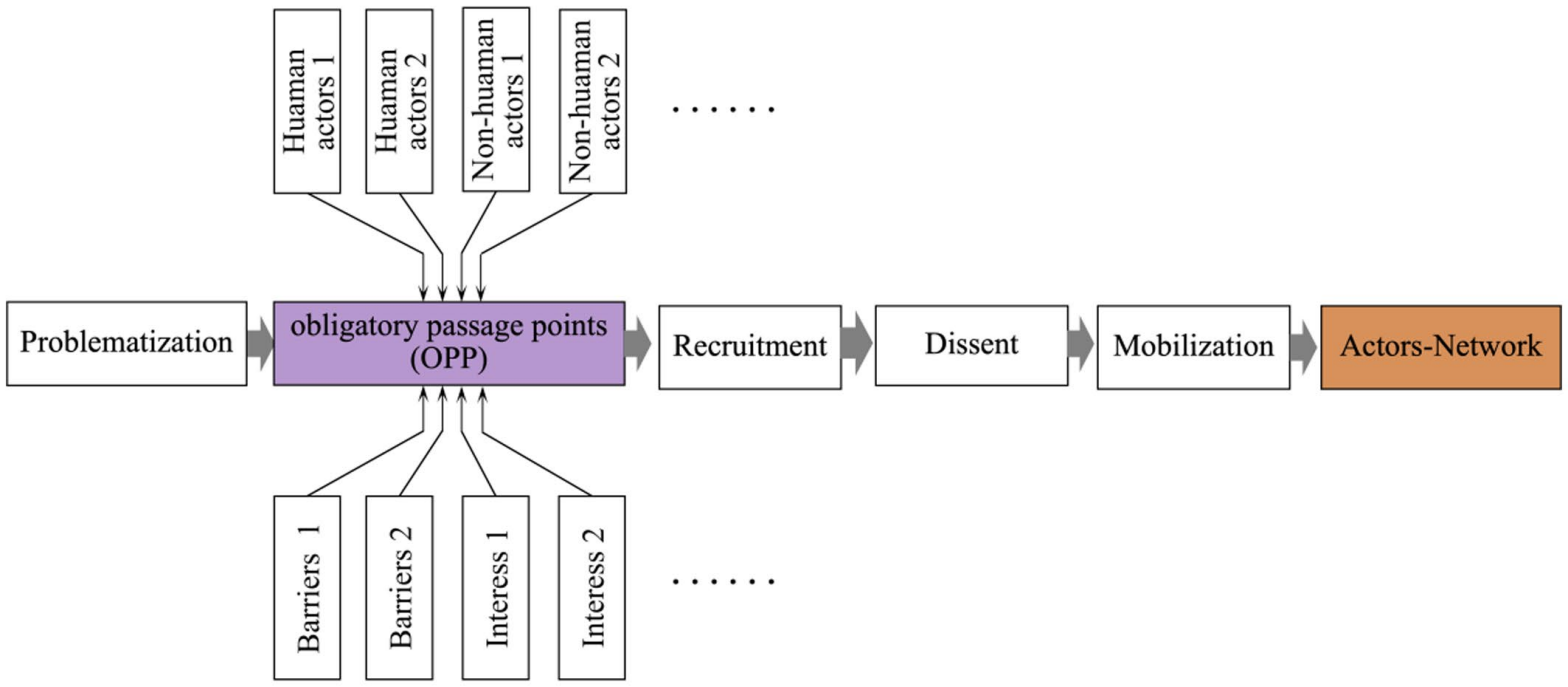
Actor network model.

Actor-Network Theory (ANT), proposed by the Paris School and further developed by scholars such as Latour (2005), provides a multi-perspective sociological analytical framework. ANT seeks to replace traditional social constructivism with the concept of practical construction, introducing relational and process-oriented thinking into scientific inquiry. Rooted in the field of Science, Technology, and Society (STS), ANT emphasizes the dynamic interactions and organic connections between human and non-human actors within evolving networks (see Figure 1), rather than attempting to uncover the underlying causes of network existence. In this framework, participants are not limited to human beings but also include non-human actors, such as texts, environments, institutions, and technological artifacts. ANT attempts to “open the black box” of science and technology by tracing the complex relationships between human and non-human elements.

Compared with other governance theories, such as stakeholder theory, agency theory, or activity theory (Miettinen, 1999), ANT provides a particularly powerful tool for revealing the complexity and dynamics of networks. According to Latour, the core elements of ANT include actors, translation, and networks. Within this framework, actors refer to any entities—human or non-human—that attempt to influence the course of a process or affect its outcomes. The agency of non-human actors lies primarily in their capacity to constrain or enable the behavior of human actors.

The formation of an actor-network is achieved through four translation processes: problematization, interessement, enrolment, and mobilization (Saito, 2011). From a methodological standpoint, ANT focuses more on “science and technology in the making” rather than “ready-made science and technology” (Latour, 2005). The main methodological approaches in ANT include following the actors and translation. The former involves interviews and ethnographic research, while the latter draws on texts and communications from various forms of media (Bilodeau et al., 2019).

According to ANT, evidence, technology, financial resources, institutions, and regulatory frameworks work together to enable theoretical innovation. Previous studies have applied ANT to explore inter-organizational integration (Chiari et al., 2023; Lee et al., 2015). However, few studies have examined the interrelationships among families, schools, and communities, highlighting the research gap that this study aims to address.

### 2.2 Literature review

Over the past century and a half, the roles of schools, communities, and families have undergone significant transformation. Schools now play a crucial role in improving students’ academic competence and physical behaviors through the establishment of collaborative partnerships (Epstein and Sheldon, 2022). In the modern era, education is no longer the sole responsibility of schools. Families, local communities, media, and new technologies all exert considerable influence on children and adolescents (Weiss et al., 2010).

In the field of PK–12 physical education, several studies have integrated school-based, family-based, and community-based physical education programs. By leveraging diverse sports resources, these initiatives aim to support students, teachers, and schools in promoting adolescent physical health and fitness (Ward et al., 2021). However, this process inherently involves cross-sectoral collaboration and multi-actor partnerships. Such collaborative mechanisms have been validated in multiple domains, including educational administration (Torenvlied et al., 2013), public health (Torenvlied et al., 2013), and health promotion (Roussos and Fawcett, 2000). Within these collaborative frameworks, relationship-building has been identified as the core of effective governance. The relationships between individuals and organizations often reveal underlying power imbalances—an essential factor that requires ongoing research to better understand how common goals can be achieved (Ceballos-Higuita and Otálvaro-Castro, 2021). Moreover, it is necessary to gain deeper insights into the diversity of public service arrangements within multi-actor collaborations and to examine varying levels of participation and integration among services, policymakers, and practitioners (Bilodeau and Potvin, 2018; Neves et al., 2021).

Additionally, the social ecological model considers how individual, interpersonal, institutional, community, and policy-level factors interact to influence health behaviors. This theoretical perspective suggests that a set of interrelated proximal and intersecting environments directly shape human developmental outcomes. Scholars such as Lawson and Lawson (2013) and Martin and Dowson (2009) argue that proximal social contexts—such as family, school, and community environments—are directly associated with students’ engagement levels. As Martin and Dowson (2009) proposed, a positive social environment fosters greater participation in physical activities. The benefits for students’ physical and mental development are maximized only when these proximal environments interact synergistically (Bronfenbrenner and Morris, 1998).

Previous research has also shown that adolescents with higher levels of social capital within school and family contexts tend to perform better in both academic achievement and physical performance (Crosnoe, 2004). Therefore, integrating family and community partnerships into school-based physical education has been identified as one of the best practices for after-school and youth programs (Coatsworth and Conroy, 2007).

In summary, existing studies consistently highlight that effective school–family–community (FSC) collaboration plays a pivotal role in enhancing adolescent physical health and fitness, reinforcing the importance of multi-sectoral cooperation for youth health promotion and holistic development.

### 2.3 Theoretical framework

The pure application of ANT remains relatively uncommon and has sparked considerable debate (Doolin and McLeod, 2005). Rather than engaging in theoretical controversies, this study focuses on examining the practical value of the ANT approach in promoting adolescent physical health and fitness within the FSC context. To illustrate this applicability, we draw upon empirical studies that have applied ANT in the fields of public health and education (Finn-Stevenson, 2014; Chiang et al., 2015), as well as our ongoing research work.

In this study, we reflect on ANT’s potential contributions and its practical relevance to the enhancement of adolescent physical health and fitness through FSC collaboration.

First, ANT provides a powerful lens for conceptualizing how different actors experience and respond to multiple realities. It enables the depiction of dynamic, interconnected relationships among actors without neglecting their interdependencies. This is particularly significant in rapidly evolving and continuously changing educational contexts—especially in government-led reform initiatives and the shifting power relations they generate (Robertson et al., 2010).

Second, from a governance perspective, the youth physical health network embodies the principles of polycentric governance. Guided by ANT, the translation process helps clarify the responsibilities of key stakeholders and delineate the interactions among diverse governance models, thereby maximizing long-term governance efficiency. ANT thus assists in mapping how various actors enact and negotiate different realities, offering a nuanced understanding of their relational dynamics—an aspect that becomes crucial when analyzing educational systems undergoing institutional transformation.

Finally, incorporating non-human actors—such as policies, financial resources, technological tools, and institutional structures—into the analytical framework broadens the research scope. This inclusion highlights the influence of non-human factors on the effective functioning of collaborative networks, demonstrating that these elements are not merely contextual but actively shape the sustainability and outcomes of FSC-based initiatives aimed at improving adolescent physical health and fitness.

## 3 Research design

### 3.1 Sample selection

In the framework of Actor-Network Theory (ANT), qualitative research serves as the primary method of data collection. Unlike hypothesis-testing research, qualitative inquiry is more appropriate for theory development, as it shifts analytical attention toward meaning-making activities, negotiations, the perspectives of different actors, and emergent effects (Cresswell et al., 2010).

This study follows Patton’s (1990) principle of purposeful sampling, which emphasizes the selection of participants based on the criterion of “maximizing information richness.” Accordingly, the sample design aligns with the research objectives and focuses on key groups involved in the collaborative mechanisms of the Family–School–Community (FSC) network. The purpose is to explore both the successful experiences and obstacles encountered during the implementation of these linkage mechanisms.

To ensure the credibility and representativeness of the findings, heterogeneity sampling was applied to include families from different economic backgrounds, schools of various sizes, and communities with differing resource levels.

The selection of individual participants followed explicit inclusion criteria:

- Parents were required to have children enrolled in the selected schools and to have participated in collaborative physical activity programs;
- School staff included physical education (PE) teachers and principals responsible for school sports programs;
- Community workers had to be directly engaged in community sports or youth development affairs;
- Government officials were required to have participated in the design or implementation of youth physical health policies;
- Students were aged between 10 and 19, possessed basic verbal communication skills, and regularly participated in school sports activities.

Regarding sample size, the study adopted Patton’s theoretical framework for purposeful sampling combined with conceptual stratification to enhance representativeness. Based on guidelines for multi-case qualitative research—which typically recommend 15–20 participants per case to capture diverse perspectives—the estimated sample size ranged from 45 to 60 participants across three regions with varying levels of economic development: Gansu (less-developed), Zhengzhou (moderately developed), and Shanghai (highly developed).

Following the above principles, a total of 67 participants were initially interviewed, including 13 parents, 21 PE teachers, 12 community workers, 4 principals, and 17 students. To expand the diversity of the sample, additional participants from social sports organizations and government management departments were later included, resulting in a final sample of 78 participants. During the sampling process, special attention was given to the teaching levels of PE teachers and the learning stages of students; in addition, teachers not directly involved in physical activity programs were also included. The detailed structure of the interview sample is presented in Table 1.

**Table 1:** Interview sample structure.

Category	Subcategory	Interviewees	Category	Subcategory	Interviewees
Parents		PS1-13	Principals		MT1-4
PE Teachers		PET1-21	Students	Primary/Middle/High School	S1-17
Community		SMP1-12			
Sports Organizations		SSO1-3	Government	Sports / Education Bureau	SB1-8

### 3.2 Data collection

Multiple data collection strategies were employed in this study, including document analysis, participant observation, and semi-structured interviews (Bowen, 2009; Hanna, 2012), in order to gain an in-depth understanding of the phenomenon (Flick, 2014). Prior to formal data collection, a pilot test of the interview guide was conducted with five participants at a primary school in Wuhan. Based on the feedback from the pilot, several revisions were made to improve clarity and comprehension.

For instance, complex or abstract questions such as *“How do you understand the process of translation?”* were simplified to “Have you ever changed your physical activity practices due to policies or others’ suggestions?” Age-appropriate questions were added for student participants, such as whether they enjoyed participating in sports activities with their parents or teachers in the community and why. Ambiguous terms were also clarified; for example, “collaborative activities” were explicitly defined as school- or community-organized events in which families could participate.

All interviews were conducted face-to-face by trained research team members to ensure authentic and interactive communication. The study followed standardized research procedures to guarantee scientific rigor and ethical compliance. Each interview lasted an average of 40 min, while interviews with students were shortened to 25–30 min to minimize fatigue.

Additionally, regional workshops were organized in each study site to include participants from different categories of the interview sample. The workshops provided an opportunity to observe professional interactions and discussions around the key research themes. After each observation session, reflective discussions were held between the observers and facilitators to identify and analyze the dynamic aspects of the interactions. Field notes were taken systematically and analyzed as supplementary data (Phillippi and Lauderdale, 2018).

With participants’ informed consent, all interviews were audio-recorded and transcribed verbatim within 24 h of completion. The final corpus comprised approximately 103,000 Chinese characters of transcribed text, with identifying information retained for traceability and verification purposes.

### 3.3 Data analysis

The Actor–Network Theory (ANT) framework guided the construction of the data coding system and the reconstruction of interactions among actors in this study. Materials collected from the three sources of evidence were decomposed through content analysis, following a set of predefined descriptive codes: associations among actors; presence of disputes and resolution mechanisms; types of heterogeneous actors; barrier factors and interest demands; and the alignment of actors, resources, and support mechanisms.

A reflexive qualitative content analysis approach was adopted to analyze the interview data, with the assistance of MaxQDA 2020 software. The analysis proceeded in systematic steps. First, the research team collaboratively analyzed the data through an iterative and cyclical process of reading, with particular attention to the emergence of common codes. Second, researchers repeatedly read the interview transcripts to become familiar with the content and to build a holistic understanding of the material. Based on this process, a coding framework was developed to capture key analytical dimensions, including types of actors, patterns of interaction, barrier factors, interest demands, stages of translation, and forms of controversy.

Throughout the entire analysis process, the research team engaged in continuous discussion and reflection, creating and refining categories while drawing upon existing literature to consolidate and deepen emerging themes. According to Corbin and Strauss (1990), theoretical saturation is achieved when newly collected data no longer contribute to the development of new categories or insights. In this study, when coding approximately one-third of the remaining interviews failed to produce new information, theoretical saturation was deemed to have been reached.

In addition, triangulation was applied by cross-verifying interview findings with evidence from policy document analysis and participant observation. This strategy further enhanced the credibility and robustness of the research conclusions.

## 4 Results

### 4.1 Identification of actors within the network

ANT adopts a distinct epistemological and ontological stance, essentially perceiving the world as composed of networks. These networks encompass humans, objects, ideas, and concepts—all of which are regarded as *actors* within the network (Latour, 2005). Tracing the associations or relationships among these components (or actors) constitutes a central analytical activity in ANT.

To identify the key components of the network, interview data were organized according to the principles of sequence, influence, stakeholder interests, and actor agency. The analysis revealed that, beyond the primary human actors—namely parents, physical education (PE) teachers, and community sports workers—other human actors such as government administrators, staff from social sports organizations, and researchers, as well as non-human actors including policies and regulations, sports venues and facilities, funding, curricula, family sports beliefs, and media, also play crucial roles within the system (See Table 2).

**Table 2:** Actors of network.

Category of actor	Type	Network member
Core actors		Government
Key actors		Schools, families, communities
Co-actors	Human	Physical education teachers, social sports organizations, research institutions
	Non-human	Policies and regulations, curriculum content, Family concept, field equipment, funding, technology, media

### 4.2 Actor-network translation analysis

#### 4.2.1 Problematization

Within the network, core actors possess high authority and are responsible for coordinating heterogeneous actors, establishing shared goals, defining Obligatory Passage Points (OPPs), and resolving conflicts. Networks are typically initiated by these core actors, who first clarify their own problems, interests, and objectives to construct a network blueprint, and then engage with other actors in the translation process (Latour, 2005).

In the Chinese context, the social and institutional characteristics of FSC collaboration necessitate a government-centered network. The government, through institutional feedback and policy guidance, collaborates with community and other sectors to address critical issues at OPP nodes. After the translation process is completed by other participants, a stable actor-network is formed.

As one participant (MT2) stated: “The government should act as a coordinator, intervening in adolescent sports activities to reasonably allocate and coordinate resources across stakeholders.” Another participant (SSO3) noted: “The government’s delineation of responsibilities can remove barriers to entry for multiple actors participating in the promotion of adolescent physical health and fitness.” Similarly, PET3 observed: “Relevant laws *and regulations issued by the government provide sufficient time and space for school-aged youth to engage in physical activities.”*

Government authorities can leverage their organizational leadership and formal authority to mobilize resources and encourage sincere collaboration among schools, social sports organizations, and research institutions. Accordingly, in this study, the network’s OPP is explicitly defined as: “Under the guidance of national policy, aligned with family education needs, promoting the comprehensive enhancement of adolescent physical health and fitness.”

#### 4.2.2 Interessement

Interessement refers to the stage in which a core actor persuades other actors to recognize and accept the definitions and roles it proposes (Latour, 2005). Conducting barrier analysis and clarifying interest alignment constitute the primary prerequisites for constructing the FSC actor-network. The government, as the core actor, identifies the problems and obstacles currently faced by various actors and establishes these issues as the Obligatory Passage Points (OPPs) that other actors must navigate to enter the network.

In this study, thematic analysis was employed to systematically examine the interview transcripts, using an inductive approach to extract data from the macro to micro levels. Initially, open coding was applied to phenomena in the raw data, labels were assigned, semantically similar labels were merged, and concepts were refined (see Table 3).

**Table 3:** Open coding.

Initial categories	Extracts of information (initial concepts)
A1 Policy objectives and coordination failure	Ten schools in our county competed for 800,000 yuan of special sports funding, and in the end, the three “model schools” took 600,000 yuan, with the rest divided among the other schools (SB4-resource allocation conflicts); […]
A2 Resource and institutional squeeze	Nowadays, PE classes are seriously undercut. When there are monthly or midterm exams, teachers of academic subjects come and borrow time for their classes, always saying they need to “catch up.” On top of that, the playground is used for morning exercises, afternoon club activities, and is rented out to outside organizations on weekends (MT4-sports time and space being compressed). […]
A3 Cognitive bias and lack of support	Compared to physical activities, I would rather my child read more after finishing his homework (S6-fixed sports concept). […]
A4 Dual scarcity of human and material resources	Our community is considered good, with a sports instructor. Many communities do not have anyone at all, and it’s hard to do activities (SMP3-shortage of community instructors). […]
A5 Professional efficacy deprivation	It’s hard to have the energy to ensure the quality of teaching (PET9-heavy workload). […]
A6 Market access and functional blockade	Parents need personalized services like myopia prevention gymnastics and physical rehabilitation, but 90% of the institutions in the market only provide “short-term long jump training” or “middle school entrance exam rope skipping training” (PET4-single service orientation). […]
A7 Government efficacy growth	In recent years, the “Healthy Campus” program has increased the number of primary and secondary school sports facilities in our city from 65 to 89%, and the pass rate for students’ physical fitness has increased by 12 percentage points. These figures directly reflect the implementation of policies (SB3-reflecting public effectiveness). […]
A8 Interest representation: promotion of school education performance	Our school has included sports performance in the “comprehensive quality evaluation,” and the admission rate to key high schools has increased by 8% in the past 2 years. More importantly, students who are physically active show stronger concentration and stress resistance in their academic studies (MT1-promotion rate). […]
A9 Family well-being improvement	After receiving sports guidance at school, my child has become significantly more cheerful. He used to complain about headaches and insomnia, but now that he plays football for an hour every day, he sleeps soundly at night and even gets fewer colds (PS4-protecting students’ physical and mental health). […]
A10 Public service optimization	The most important safeguards for young people’s participation in sports in the community are staff organization and the provision of venues and equipment. So, we are hoping the government will step up their game and give more support to community sports (SMP4-obtaining resource support). […]
A11 Professional value realization	When we team up with other departments or folks in charge, our PE teachers’ main mission is to get students pumped about sports and boost their physical health (PET7-Improve students’ physical health). […]
A12 Market volume expansion	When we work with schools, the huge involvement of PE teachers, students, and parents has really increased our brand’s population base and expanded our business (SSO3-Improve brand influence). […]

Based on the relationships among the identified concepts, the study classified them into 12 categories through open coding. Subsequent axial coding led to the identification of two overarching categories: implementation barriers and interest factors (see Table 4). The results of the data analysis, when interpreted in conjunction with existing literature, indicate that the barriers and interests associated with various actors within the FSC network primarily include the following:

**Table 4:** Main category.

Main category	Initial category	Initial concepts
Barriers	A1 Policy objectives and coordination failure	A1 vague responsibilities and rights; A2 conflicting resource allocation; A3 difficulties in collaboration
	A2 Resource and institutional squeeze	A4 compressed time and space for sports; A5 single venues and equipment; A6 lack of physical education teachers
	A3 Cognitive bias and lack of support	A7 neglect of the value of health; A8 lack of sports guidance
	A4 Dual scarcity of human and material resources	A9 shortage of community instructors; A10 low equipment utilization rate; A11 financial difficulties
	A5 Professional efficacy deprivation	A12 lack of a voice; A13 heavy workload; A14 difficulty quantifying results
	A6 Market access and functional blockade	A15 Barriers to transfer; A16 Limited autonomy; A17 Single service orientation
Interests	A7 Government efficacy growth	A18 Demonstrate public effectiveness; A19 Drive economic growth; A20 Strengthen governance legitimacy
	A8 Promotion of school education performance	A21 Improve students’ physical health; A22 Improve school reputation; A23 Promotion rate
	A9 Family well-being improvement	A24 Ensure students’ physical and mental health; A25 Gain an advantage in promotion;
	A10 Public service optimization	A26 Obtain resource support; A27 Undertake government projects; A28 Improve resident satisfaction
	A11 Professional value realization	A29 Improve student physical fitness; A30 Title evaluation
	A12 Market volume expansion	A31 Improve brand influence; A32 Obtain income

The findings from the investigation of interactions among relevant actors enabled the identification of their local and organizational relational priorities. Through this process, several practical issues were recognized, which shaped how actors identified potential collaborators and implemented collaborative and integrated action plans aimed at promoting adolescent physical health and fitness.

As noted by Callon (2006) and Djohy (2019), collaboration can be conceptualized as a socialization process in which resources such as knowledge, skills, values, and attitudes are produced. This process depends on the policies guiding socio-technical implementation and the willingness of actors to integrate into the networks of other participants.

To establish a tightly coordinated relational network, the government must mobilize a variety of human and non-human allies, including equipment, documents, and policy or work contracts. This approach lays a solid foundation for ensuring that every adolescent included in the network has access to physical activity, along with conditions that support health, nutrition, safety, and well-being.

#### 4.2.3 Enrollment

Enrollment refers to the process by which core actors recruit neutral or potential actors into the network based on principles of diversity and relevance, thereby maintaining the dynamic adjustment of the network (Tummons, 2021). An increase in actor nodes within the network may transform weak informational ties into stronger, more formalized connections, potentially accelerating the flow of information. By clarifying specific interests, the government and schools can enlist human actors, whereas the enrollment of non-human actors involves designating qualified spokespersons and incorporating relevant organizations or individuals that can represent their interests and possess discursive authority.

Core actors can mobilize both formal and informal networks within schools and communities to promote family-centered physical activity models, including encouraging family-based sports and demonstrating respect for family concerns (Mancini et al., 2007). Communities should attract volunteer parents, especially those with sports expertise, to establish healthy volunteer resources and coaching teams that support adolescent health education (Willgerodt et al., 2021). Moreover, acknowledging and leveraging the unique beliefs families bring to schools and communities is a critical step in fostering a school environment that values family engagement.

When schools empower families to take leadership roles within the school-community network, families can further mobilize their own networks, enhance connectivity, and increase both participation and engagement. Examples include inviting families to join Parent-Teacher Organizations (PTOs), sharing practical experiences, providing direct feedback on school policies based on family knowledge and life experience, and establishing peer support mechanisms where capable families assist those in need—these strategies effectively enhance family participation in school affairs.

Schools should actively collaborate with sports and health organizations and committees to seek guidance on nutrition, exercise methods, and chronic disease management. They can also establish Community Health Promotion Specialist (CHPS) positions to support schools in implementing physical activity standards. These organizations and personnel serve as intermediaries among families, schools, and communities, effectively complementing the work of educational and sports departments in promoting and implementing adolescent health initiatives. For example, the Arkansas legislature established the State Children’s Health Advisory Council (CHAC) to formulate school nutrition and physical activity regulations, advise the Arkansas Department of Education and Health, and mandate that regional Community Health Nurse Specialists (CHNS) collaborate with schools on physical activity initiatives (Hills et al., 2015). The council’s responsibilities include convening regular team meetings, raising awareness and support for school-based physical activity, generating and organizing implementation strategies, securing resources and coordinating fundraising, and overseeing program monitoring and evaluation. Its core mission is to guide and facilitate partnerships and collaboration between schools’ physical activity programs and key local stakeholders—including administrators, faculty, parents, and community residents.

To promote collaborative governance across family, school, and community networks, governments and schools should relax restrictions and create platforms for external social actors, such as sports associations and fitness training organizations, to participate. This can be achieved through direct measures such as financial subsidies, tax incentives, facility rental support, loans, and naming sponsorships (Carson et al., 2014). Additionally, schools can decentralize authority via competitions or commissioned services to identify qualified sports associations, thereby enriching after-school physical activity programs, alleviating the financial burden on families, schools, and communities, and meeting the growing demand for high-quality, professionalized after-school sports services.

#### 4.2.4 Dissent and mobilization

Actor networks are influenced by spatiotemporal factors, environmental variables, and the characteristics of both implementing subjects and objects. In the construction and operation of networks, issues such as improper decision-making and unclear task allocation inevitably arise (Tummons, 2021). Therefore, the dissent and mobilization stage is a critical part of the translation process, serving as a key mechanism to reconcile the interests of multiple actors and ensure the sustainable development of the actor network.

The FSC network exhibits dynamic and adaptive characteristics, comprising diverse actors, each possessing distinct resources and interests. Such diversity inherently generates various conflicts and tensions during network operations. Drawing on existing studies and comprehensive network surveys, we propose five potential dissent types that may emerge in the operation of heterogeneous actor networks in future development. Specifically, these questions include: (1) how to balance the interests of all actors under dynamic network conditions; (2) how to achieve balanced development between core operations and thematic activities of heterogeneous actors; (3) how to maintain regular and efficient communication among families, schools, and communities; (4) how to guide and regulate family-based physical activity; and (5) how to assess the effectiveness of multi-party parenting and cross-sector collaborative governance.

To address these challenges, heterogeneous actors should actively mobilize, continually coordinating the visions and actions of different stakeholders. First, incentive and safeguard mechanisms translate theoretical objectives into tangible outcomes. These can take the form of material rewards, signed agreements, promotions, professional title evaluations, democratic voting, or target-based incentives (Wang et al., 2024), ensuring the sustainable operation of the interest network. Through standardized safeguard documents, mutually beneficial actor alliances are cultivated, enabling primary educational entities to perceive a sense of achievement and fulfillment in their roles.

Second, with the technical support of artificial intelligence, big data, and other network technologies, cross-sector electronic collaboration platforms can be established to stabilize the actor network. Such platforms break down temporal and spatial barriers in educational communication, allowing families, schools, and communities to collaborate and share information in real time. By integrating internal and external resources, these platforms facilitate the collection and sharing of educational resources and promote multi-dimensional participation in offline physical activity programs. For example, in Colorado, the “Healthy Schools Smart Resources” tool was developed for district- and school-level assessment and implementation of health policies and practices, funded by Kaiser Permanente and in collaboration with the local Department of Education, Public Health and Environment, and the Healthy Schools Alliance (Piekarz-Porter et al., 2017). This initiative integrates school health assessment questions statewide, simplifying data collection and submission, and aims to provide actionable data and best practice guidance for health and well-being.

Stricter legislation has been associated with improved student physical activity levels in public schools (Mâsse et al., 2013). As core actors, governments and relevant departments need to proactively implement policies and safeguards to facilitate the rapid establishment of the FSC network. For instance, Act 180 facilitated a state-level joint-use funding program operated by the Arkansas Department of Education (ADE), Arkansas Department of Health (ADH), and the Arkansas Center for Health Improvement. This program assists schools in adopting and implementing shared-use policies and establishes collaborative community partnerships to increase opportunities for physical activity (Piekarz-Porter et al., 2017).

Moreover, the FSC network should develop Family Physical Activity Guidelines to standardize and promote family-based physical activities. This creates a supportive, harmonious, and health-promoting family environment. Schools and communities can also organize regular activities, such as family school visits, sports workshops, and parent–child physical activities, allowing parents to participate in PE classes with their children and learn physical skills from the child’s perspective.

Finally, regular monitoring and evaluation must be strictly adhered to. Implementation evaluations ensure the establishment of high-quality, sustainable FSC partnerships (Epstein and Sheldon, 2022). Educational and sports departments should establish appropriate internal oversight mechanisms to create a closed-loop actor network. Specific FSC evaluation criteria include (Dove et al., 2018; Zhu et al., 2025): (1) coordination of school health work; (2) family and student participation assessment; (3) health education evaluation; (4) health service assessment; (5) nutrition assessment; (6) physical education/physical activity evaluation; (7) staff health assessment; (8) school counseling, psychological, and social work services evaluation; (9) school and community environment evaluation; and (10) funding support assessment. Evaluation should prioritize health outcomes and combine qualitative and quantitative methods to identify issues and propose improvements.

#### 4.2.5 Network formation

**Figure 2:**
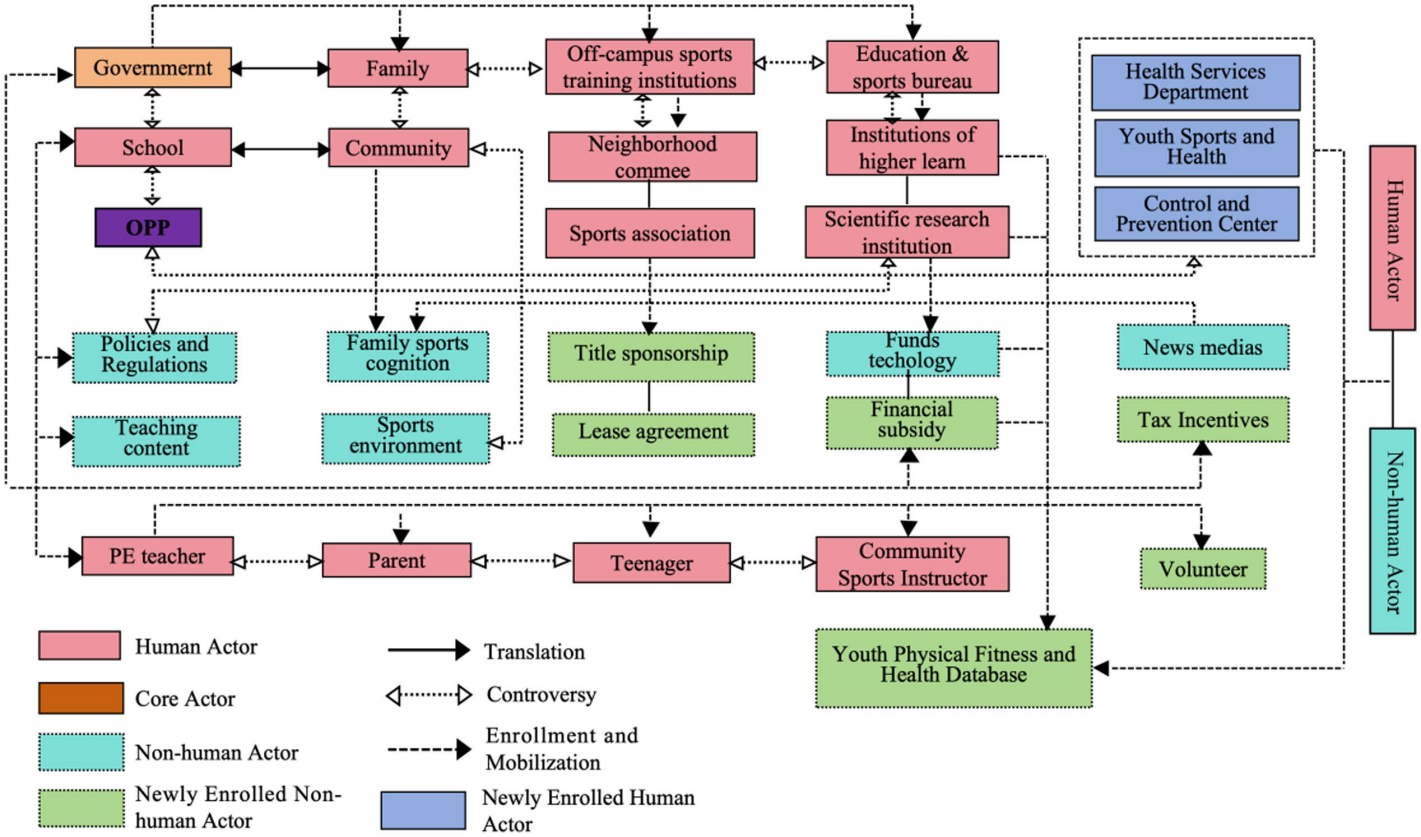
Actor network for promoting adolescent physical health and fitness.

By integrating the four translation processes outlined in the previous sections, a final actor network model was established (see Figure 2, Multi-Actor Collaborative Network for Promoting Adolescent Physical Health and Fitness). The model includes the following key mechanisms: (1) Core actors supervise the establishment of the Obligatory Passage Point (OPP), encourage participation from other actors, coordinate diverse thematic activities, recruit and mobilize new actors, and resolve dissent; (2) Heterogeneous actors, such as government entities, simultaneously work toward a shared vision while constructing effective collaborative platforms. These platforms facilitate the translation of each actor’s interests, thereby laying the foundational conditions for collaborative network governance, mobilizing new actors, and addressing oppositional perspectives; (3) Beyond forming alliances with other heterogeneous actors, governments and schools must manage tensions between the OPP and the interests of other actors. Qualitative analysis indicates that many school-led collaborative parenting or co-education activities tend to focus on internal operations, which may weaken the school’s physical education functionality and professional capacity. Balancing these tensions constitutes a significant challenge that the entire actor alliance must carefully consider; (4) Actor-Network Theory (ANT) does not predefine the world as micro or macro environments, nor does it ascribe agency exclusively to individuals or social structures. Broader environmental factors—including political, cultural, and economic contexts—should be regarded as integral components of the network. These non-human actors must be considered during the introduction of interventions or software systems, and require sustained support to ensure the network’s effective operation.

## 5 Discussion

The core findings of this study regarding the FSC linkage network are not only highly consistent with the fundamental principles of global multi-actor collaborative governance theory but also demonstrate context-specific adaptive characteristics in practical implementation. As noted, Chen et al. (2019) confirmed in a study of interventions for Chinese adolescents’ physical activity that cross-sector integration of school, community, and family sports resources is significantly positively associated with adolescents’ health behaviors and psychological well-being.

First, this study identified multiple heterogeneous actors participating in the FSC network, including human actors such as government officials, schools, and teachers, as well as non-human actors such as funding, policies, and equipment. However, collaboration and integration among these actors face multiple challenges within the network: bureaucratic barriers, resource scarcity, and cross-organizational policy misalignment (Borvil et al., 2021). This aligns with the obstacle factors identified in our coding analysis, including shortages in policies, venues, and resources, as well as unclear responsibilities. Similar research suggests that reduced public funding, high registration fees, and unsafe facilities are significant barriers to children’s participation in quality sports programs (Wicks et al., 2007). While strong network leadership could potentially mitigate these challenges (An et al., 2017), it is important to note that actor-network theory (ANT) emphasizes decentralization; strict hierarchies are not inherent. Instead, actors tend to cooperate and build partnerships within the network rather than operate individually (Meisel et al., 2014), sharing information, values, and norms, and assisting each other in problem-solving. Consequently, organizations within the network must form cohesive factions based on funding and resource support (Barnes et al., 2010).

Moreover, this study defined non-human actors, such as policies and regulations, sports facilities and equipment, and family health cognition, as key nodes in the network. This perspective extends the analytical dimension of existing international research (Hobbs et al., 2023; Slimmen et al., 2024). For instance, community and school environmental factors are important determinants of insufficient adolescent physical activity; however, prior studies often treat them as passive contextual variables. Using ANT, this study interprets these non-human elements as active actors capable of constraining or facilitating behavior. This aligns with Sayes (2014), who argued that non-human entities can actively shape human behavior logic and serve as central influence factors in network operations.

The study also revealed that the network dynamically evolves both within and between organizations. This raises critical questions: Are adolescent health behaviors changing? Is the network itself evolving? Research on the mobility of physical activity (PA) networks (Timm et al., 2021) suggests that such dynamics may challenge network stability and impede the influence of other organizations. Therefore, future actor-network analyses should focus on addressing longitudinal changes in PA implementation.

Finally, this study highlights contextual specificities in China. The government plays a dominant role within the FSC model, and the “strong government, weak society” institutional characteristic shapes the governance structure of the network. However, collaboration within the network tends to prioritize meeting school accountability requirements rather than establishing genuine learning partnerships with students (Pang, 2011). Consequently, parental involvement can build social capital through school-family cooperation, while the government and schools retain primary responsibility for organizing educational activities.

## 6 Limitations

Although this study carefully applied actor-network theory (ANT) to analyze the relationships among government, families, schools, and communities in China, several limitations and shortcomings remain. First, the study primarily relied on interview data and relevant policy documents for actor-network analysis, lacking quantitative support—a common limitation in ANT research (Cresswell et al., 2010). Second, the theoretical approach is largely descriptive and does not provide detailed guidance on how to conceptualize actors, analyze their behaviors, or interpret findings. Moreover, the agency and power distribution of non-human actors were not fully examined (Sayes, 2014), and the cross-sectional design limits the ability to capture the network’s dynamic evolution. Third, the identification of core actors and the translation steps outlined in this study were not evaluated for practical effectiveness, leaving the operational outcomes of the network unverified. Finally, the study’s scope was geographically limited, with some participants drawn from underdeveloped regions. These areas often have fragile service networks, which may result in unstable network nodes, and such phenomena are difficult to quantify.

Future research can advance the field by providing a comprehensive description of all relevant interactions. Nationwide, multi-center, and international comparative studies of FSC models, employing mixed-methods approaches, could quantitatively assess the network’s positive impact on *adolescent physical health and fitness*, and extend findings beyond China. Combining ANT with power theory could further illuminate power dynamics and interest negotiations among actors, while calculating cost–benefit ratios for various strategies could offer empirical evidence for policy optimization. Regarding the methodological limitations of ANT, researchers should avoid focusing exclusively on fine-grained network details at the expense of broader research objectives. It is also essential to recognize individual differences among human actors and acknowledge that artifacts possess inherent attributes and historical trajectories that shape network interactions.

## 7 Conclusion

The growing demand for improvements in adolescent physical health and fitness highlights the necessity of a more nuanced understanding of relationships across different sectors. In this context, actor-network theory (ANT), with its radical conceptualization of agency and the relationships between humans and non-humans, has stimulated significant academic discussion. This study illustrates how ANT can effectively guide research assessing adolescent physical health and fitness within family, school, and community environments, and offers recommendations on establishing and maintaining such networks. The primary value of this approach lies in its capacity to capture the fluidity and complexity of multiple actors, to recognize the active role of material entities in shaping social relations, and to provide theory-driven guidance for sampling and data collection.

Furthermore, our findings emphasize the necessity of embedding local participants, resources, and structures into the network. The importance of sustained and open communication and information management among stakeholders is evident. By discussing, negotiating, and rearranging these components in a participatory manner, cross-sector collaboration can be supported, thereby promoting health enhancement and equitable impact. When seeking coordinated actions within integrated mechanisms to advance adolescent physical health and fitness, the need for ongoing commitment becomes clear. In this way, professionals and administrators engaging in a network oriented toward youth sports development can profoundly influence family practices and adolescents’ physical health outcomes.

Social network analysis (SNA) provides unique insights within health promotion networks, revealing how families, schools, and communities collectively promote youth physical activity—insights that are not readily obtainable through traditional surveys and statistical analyses (An et al., 2017). ANT represents a promising approach, offering benefits not only for research but also for the promotion of youth sports participation and structural interventions. Interdisciplinary collaboration across sectors creates opportunities for successful and sustainable health interventions and fosters positive lifestyle behaviors, ultimately enhancing both the physiological and psychological well-being of adolescents.

## Data Availability

The raw data supporting the conclusions of this article will be made available by the authors, without undue reservation.
