# Correction: The family, school and community linkage of improving adolescent physical fitness and health: based on the ANT

**DOI:** 10.3389/fpsyg.2025.1760893

**Published:** 2025-12-17

**Authors:** Yangyang Liu, Qiuhan Zhu, Shuqing Li, Linhan Yao, Duan Wang

**Affiliations:** 1 School of Kinesiology and Physical Education, Zhengzhou University, Zhengzhou, China; 2 Student Services Department, Hainan Lausanne Tourism University, Sanya, China

**Keywords:** Actor-Network Theory, adolescent physical fitness and health, family school and community, synergetic net, school physical education

Author Qiuhan Zhu was erroneously omitted as equal contributing author.

The original version of this article has been updated.

